# The STORK dataset: Linked midwifery and delivery records of the mothers and index children in the Avon Longitudinal Study of Parents and Children (ALSPAC)

**DOI:** 10.12688/wellcomeopenres.16247.1

**Published:** 2020-10-05

**Authors:** Mark Mummé, Andy Boyd, Jean Golding, John Macleod

**Affiliations:** 1 Avon Longitudinal Study of Parents and Children, Population Health Sciences, Bristol Medical School, University of Bristol, Bristol, Bristol, BS8 2BN, UK; 2 Department of Population Health Science, Bristol Medical School, University of Bristol, Bristol, Bristol, BS8 2BN, UK; 3 Centre of Academic Child Health, Bristol Medical School, University of Bristol, Bristol, Bristol, BS8 2BN, UK

**Keywords:** Maternity records, delivery records, birth records, obstetric data, record linkage, longitudinal study, cohort study, ALSPAC

## Abstract

This data note describes the linked antenatal and delivery records of the mothers and index children of the Avon Longitudinal Study of Parents and Children (ALSPAC) birth cohort study. These records were extracted from the computerised maternity record system ‘STORK’ used by the two largest NHS trusts in the study catchment area. The STORK database was designed to be populated by midwives and other health professionals during a woman’s pregnancy and shortly after the baby’s birth. These early computer records were initiated in the early 1990s, shortly before the start of enrolment to ALSPAC. At this time the use of electronic medical record systems such as ‘STORK’ was very new, the accuracy of the records has been questioned and little contemporary detailed documentation is available. Small sample spot checks on the accuracy of the information in ‘STORK’ suggests extensive missingness and differences against gold-standard fieldworker abstracted information in some variables; yet high levels of completeness and agreement with gold-standard data in others. Software code was created using STATA (StataCorp LLC) to transform the original CSV (comma-separated values) files into a cohesive and consistent format which was reviewed for data-completeness for its potential use in future research. The cleaned ‘STORK’ records provide health, social and maternity data from the very earliest period of the ALSPAC study in an easily accessible format, which is particularly useful when other sources of data are missing.

## Introduction

The Avon Longitudinal Study of Parents and Children (ALSPAC) is a multigenerational birth cohort study which aims to compile a rich databank containing information on participants’ health and social exposures and subsequent outcomes across the life course, Within this there is a need to characterise pregnancy exposures and birth outcomes in order to inform investigations relating to the developmental origins of health and disease. ALSPAC aimed to recruit participants as early in pregnancy as possible in order to capture information prospectively, at key timepoints, and to collect biological samples during pregnancy. Information about the mother, foetus and child were collected from the mother and the baby’s father/mother’s partner using self-reported questionnaires. To complement the self-report data, ALSPAC fieldworkers have abstracted a sub-set of contemporaneously recorded clinical observations contained within the paper maternity and birth records which were compiled during the mother’s antenatal care and the delivery of the child ^1, 2^. This Data Note describes data provided via record linkage from the ‘STORK’ electronic midwifery and delivery records. These records were aimed to be compiled during the mother’s pregnancy and at the point of delivery in parallel to the paper maternity and birth records. This note also describes the birth outcome (delivery, stillbirth and death) notification lists that ALSPAC received on a weekly basis from the local NHS during the study’s initial recruitment period (1991–1992) and beyond into 1993.

The STORK data set includes information on antenatal tests, medication and pain-relief, key dates, gestational age, the placenta, labour, delivery and birth measurements. In addition, STORK includes information on previous pregnancies and patient socio-demographic information about the mother and father/partner. The STORK data can be linked with ALSPAC self-reported data, assayed biological samples, abstracted clinical notes and used for quality triangulations and to inform missing data strategies. It is worth noting that the STORK system was a very early electronic system within the NHS and there are quality issues with the data, which are described in this note.

The birth outcome lists contain a subset of STORK data, largely comprising personal identifiers, and are therefore not available as a research data file.

## Materials and methods

### The ALSPAC sample

The Avon Longitudinal Study of Parents and Children (ALSPAC) is a prospective population-based study. Initial recruitment of pregnant women took place from September 1990- December 1992 inclusive, and the health and development of the index children from these pregnancies and their parents have been followed ever since. Within ALSPAC the original pregnant women and their partners are referred to as Generation Zero (G0) and the index children as Generation One (G1).

ALSPAC recruited 14,541 G0 pregnant women who were resident in Avon, UK (former county covering Bristol and the surrounding areas in the South West UK) with expected dates of delivery 1st April 1991 to 31st December 1992. Of these initial pregnancies, there were a total of 14,676 foetuses, resulting in 14,062 G1 live births and 13,988 children who were alive at 1 year of age ^3, 4^. The eligible sampling frame was constructed retrospectively using linked recruitment and health service records. Additional offspring that were eligible to enrol in the study have been welcomed through major recruitment drives at the ages of 7 and 18 years; and through opportunistic contacts since the age of 7. A total of 913 additional G1 participants have been enrolled in the study since the age of 7 years with 195 of these joining since the age of 18 ^5^. This additional enrolment provides a baseline sample of 14,901 G1 participants who were alive at 1 year of age.

### The STORK database

**Figure 1.**
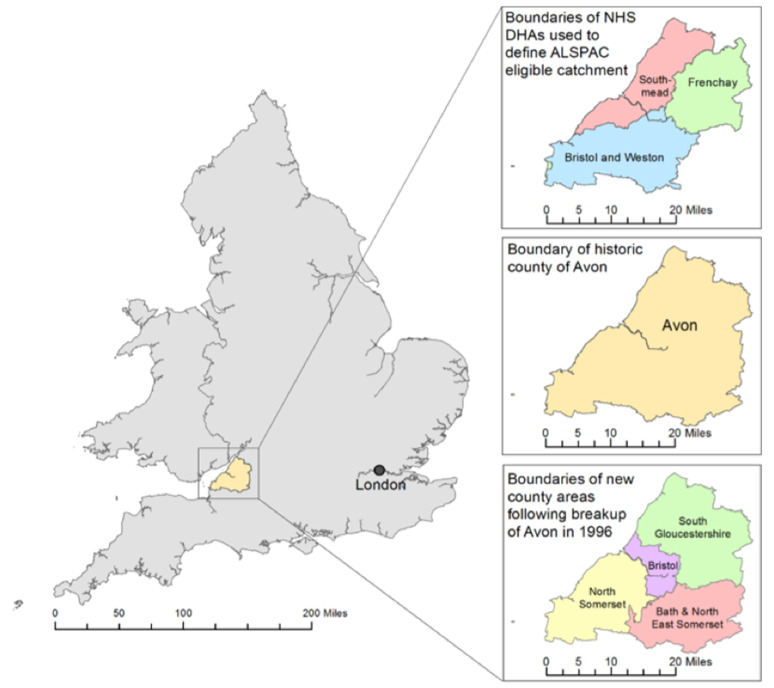
The ALSPAC Eligible Study Area within the UK. This illustrates the NHS District Health Authorities (DHAs) used to define: the ALSPAC catchment area; the historical county of Avon; and the four authorities formed following the breakup of Avon. Contains Ordnance Survey, Office of National Statistics and National Records Scotland data © Crown Copyright/database right 2014.

In January 1990 the South Western Regional Health Authority (SWRHA) Computer Centre introduced a new electronic medical record (EMR) system – called STORK - designed to capture maternity and delivery information. The name is not an acronym and was selected due to the folk tales regarding Storks (a species of bird) delivering babies. The STORK system was deployed across the SWRHA area. After an initial pilot at Bristol Maternity Hospital the other sites were converted over to the new system. The ALSPAC geographic catchment area is a geographical subset of SWRHA, having been made up of the old catchment areas of the three NHS DHAs (District Health Authorities) of Bristol and Weston, Frenchay and Southmead. The catchment area is shown in Figure 1 together with the current county boundaries for context. The system was introduced before the start of the ALSPAC recruitment campaign and over nine months before the first index child was born: meaning, in theory, the full antenatal records should be available for the majority of study participants.

The STORK system was planned to follow the course of the pregnancy from ante-natal care through to the puerperium. Following a ‘booking’ consultation in primary care (the first official registration of the pregnancy with the health system) the pregnant woman would make an appointment with her allotted midwifery team. At the first registration visit the woman’s demographic details and health details were recorded. Additional information was recorded about the father/partner who was potentially present. The intention was to type the information directly into the system, although the adherence to this is unclear given it was a new system being used in an era where many professionals were not familiar with computers and databases. The information is typically captured in coded values or constrained fields. Free text fields allow the capture of information not covered by the standard codes, as outlined in the STORK user’s manual (see *Extended data*, Supplement III ^6^) and permitted the midwife to enter anything they felt was relevant.

The system was designed to link with SWRHA’s inpatient systems (the system used in hospitals to record details on those receiving care and staying overnight). Admission information is entered on the hospital’s STORK Maternity computerised database system and the relevant details copied to the inpatient system. Transferring and discharging was also done from within the STORK Maternity system. There is a link between STORK and the SWRHA Child Health database ^7^: which was used to track early-years child development and routine healthcare (e.g. vaccinations, school nurse visits). This transfers the Birth Notification and relevant discharge information to the Child Health computer.

At discharge from maternity services the Midwife was prompted to enter more information about the care of the mother and her baby/babies. From this a wide range of discharge prints and standard statistical reports could be produced.

The STORK maternity system was piloted at the Bristol Maternity Hospital (BMH) which was one of two teaching maternity hospitals in the ALSPAC catchment area. The ante-natal side of the system went live at the start of August 1989 and the Labour/Delivery part of the system was to follow from January 1990. Other sites, including the other two maternity centres in the ALSPAC catchment area at Southmead (in North Bristol) and Weston General Hospital (Weston Super Mare), were intended to be converted over to the new system and were hoped to be fully live by the first of April 1990, although the exact dates are no longer available.

### The ALSPAC STORK dataset

A STORK data report was extracted for ALSPAC by the SWRHA Maternity Team Leader. Extracts of the St Michael’s records were taken on 04/11/1992 and the Southmead data was taken on 07/10/1992. This means they are not complete and miss later antenatal and delivery information (ALSPAC’s index generation have birth dates extending into early 1993). Due to a lack of permissions, the records from Weston hospital were not obtained.

The data selection criteria were based on the ALSPAC eligibility criteria, selecting women with an Expected Date of Delivery (EDD) between 1 ^st^ April 1991 and 31 ^st^ December 1992. The EDD used by ALSPAC to define eligibility, was done on the last menstrual period (LMP)– not the clinical estimate and reported by the mother when enrolling. This contrasts with the best-guess gestation usually used by ALSPAC currently ^1^, which is calculated by the obstetric team (usually by ultrasound) where available or by LMP date if no obstetric date was given. Pregnancies resulting in early foetal loss (≤20 weeks gestation) and home deliveries were excluded unless the baby or mother were admitted to the hospital within 28 days of delivery. Information was extracted on all individuals present in the system between these EDDs regardless of enrolment status in ALSPAC. Some of the entries relate to pregnant women who lived outside of the ALSPAC catchment area but received treatment or gave birth within the area, and also records of women who lived and received treatment within the ALSPAC catchment area and subsequently gave birth outside of this area.

### Birth outcome lists

Separate from the STORK data (but potentially generated from the STORK system, or the STORK-fed child health database) were the ‘birth outcome lists’ provided to ALSPAC on a weekly basis by local NHS staff. These print outs systematically listed all deliveries (including stillbirths) and death notifications to inform the study that an enrolled participant had given birth and to subsequently trigger a Congratulations card and the postnatal data collection. The print outs contained the following information: 1) the date of the print out; 2) mothers surname, date of birth and hospital ID number; 3) the child’s surname, date of birth and the Child Health system ID number (‘SYSNUM’); 4) the child’s sex, birth status (live, stillborn, subsequently died), gestational age (in weeks), date of death, hospital of birth; 5) home address; 6) birthweight (in grams) and birth order (where a multiple delivery); 6) mothers forename(s) and details on her previous pregnancy outcomes (number of liveborn deliveries, stillborn deliveries, aborted deliveries). Importantly the notification of a stillbirth or neonatal death triggered: (i) a letter of condolence from ALSPAC; (ii) ensuring that no further standard ALSPAC questionnaires were sent to the mother, and (iii) an invitation to take part in a study of perinatal deaths.

The Child Health system ID number is unique per child. It is an eight-digit number with no checksum. The delivery lists did not contain the national NHS ID number (which at that time were not allocated at birth ^8^ and had substantial quality issues).

The Birth Outcome Lists are paper print outs and have never been keyed. There are no structured data describing the quality of the linkage, although there are some annotations on the original paper records. These original paper Delivery Lists are maintained in secure conditions within the ALSPAC Data Safe Haven.

### Linkage to the ALSPAC database

Study staff manually matched the Birth Outcome Lists, and SYSNUM, to entries on the ALSPAC administrative database. The record linkage was achieved through structured querying of the database (i.e. software code that searched a database in a consistent and structured manner) on a case-by-case basis using deterministic approaches using personal identifiers with high levels of discriminatory power (mothers forename, family name, date of birth, full address, date of delivery, child sex, birth order). Where ambiguities remained, manual checks involving birth weight, hospital name and gestational age were also used.

This linkage between the ALSPAC database and SYSNUM was later used to link the STORK database to the ALSPAC database. Within this, SYSNUM was matched deterministically, with date of birth and child sex also being used as matching variables.

### The STORK research file: denominator

The STORK research file has been filtered to exclude information on participants who have not enrolled into ALSPAC and those who have subsequently objected to the study’s use of their linked NHS records. Of an original (provided) total of 18,444 records, 17 were dropped as they were not eligible to participate in ALSPAC, leaving a possible cohort from STORK of 18,427. Although eligible at birth a further 4,922 records were removed as they did not enrol.

**Figure 2.**
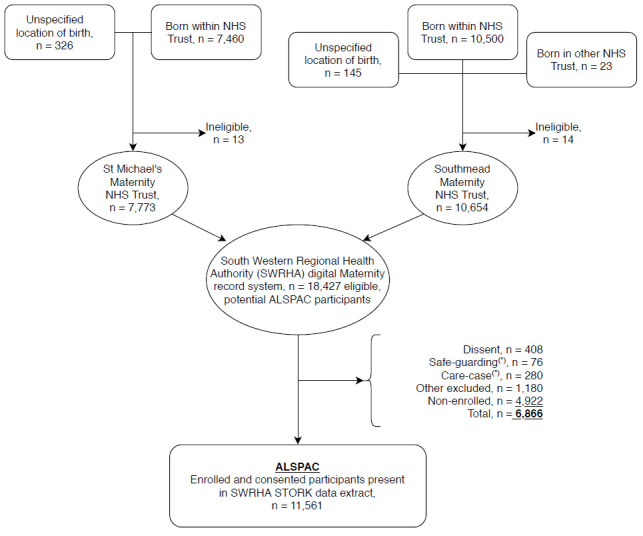
STORK dataset flow diagram. *The terms ‘safe-guarding’ and ‘care-case’ were sometimes used in the early days of ALSPAC to indicate that automatic contact, or any contact, could have been inappropriate and manual review was required. Due to this lack of clarity, they have not been given the opportunity to consent since the advent of GDPR and so have been excluded here.

A further 1,944 records were subsequently removed due to permissions statuses and other factors resulting in a loss of contact over time (n=1,180). These permission statuses include such issues as participants who dissented and withdrew from the study (n=408), study families whose circumstances mean that the study were not able to establish linkage permissions (e.g. the G1 child permanently lacks the capacity to consent, the family have requested a break from study contact) (n=280 + 76). This leaves a working sample of pregnancy records including 11,357 G0 mothers and 11,561 G1 offspring ( Figure 2). This represents 90.4% of the 12,792 G1 participants still enrolled in ALSPAC and for whom ALSPAC have permission to access their health records. These numbers are correct at the time of writing. However, the numbers making up the exclusions can be dynamic; for example, as participants change their enrolment or linkage permission status.

### The STORK research file: data processing

The dataset was provided as a series of basic text files of the raw data as recorded by STORK. There are 476 variables per delivery contained within five tables detailing information about the baby (the ALSPAC G1 index participant) and eight 8 tables detailing information about the pregnancy (the ALSPAC G0 mother). All 13 tables contained an internal ‘StorkLink’ ID unique to each pregnancy plus a ranking (birth order) for a multiple pregnancy. The ‘Storklink’ value was used to combine the information and was subsequently dropped once the tables were linked.

An excerpt showing a list of data items collected, taken from a contemporaneous users’ manual from 1989 (see *Extended data*, Supplement III ^6^), provided the basis of the information needed to map data codes into a labelled format.

The original dataset contained a great deal of patient identifiable data which needed to be either removed or anonymised in such a way as to minimise the loss of data which may be useful for research (see Table 1).

**Table 1.** Data variables which have been modified, removed or are subject to restricted distribution due to the disclosive nature of the original data.

Variable Name	Processing
Currstateofinfanttext	this contains free text including some names of siblings from previous pregnancies, which is potentially disclosive and so not permissible for public distribution, removed.
Dateofbirthofmum	Full Date of birth (DD/MM/YYYY) converted to month and year of birth (MM/YYYY).
Dateofdelivery	altered to show month and year only.
Lmpdate	altered to show month and year only.
Eddbyobstetrician	altered to show month and year only.
Dateofonsetofregcont	altered to show month and year only.
Deliverydischargedate	altered to show month and year only.
Eddbylmpdate	altered to show month and year only.
Maternitynumber	unique identifier used at NHS trust, removed.
Mothersethnicgroup	protected characteristic, restricted distribution.
Fathersethnicgroup	protected characteristic, restricted distribution.
Dateofruptofmembranes	altered to show month and year only.
Babysdischargedate	altered to show month and year only.
Dateofbirthofbaby	altered to show month and year only.
Dateofdelofplacenta	altered to show month and year only.
Babyshospitalnumber	unique identifier used at NHS trust, removed.

Although some dates have been truncated, the STORK dataset also contains derived values of gestation, length of labour and the interval between membrane rupture and delivery (as derived within the original STORK system).

**Dataset** – the STORK data provides information on:

Previous pregnancies

Table 2 describes the level of completeness of data available from the 7,637 records of G0 mothers who had had a previous pregnancy. Only those G0 mothers with a previous pregnancy recorded are included. The level of completeness of the data is given as a percentage and only indicates how many records have some data recorded but does not indicate accuracy or quality of that data. Previous pregnancies beyond the 10 ^th^ have been combined for the reason of small numbers.

**Table 2.** Completion numbers of STORK variables available describing the recorded previous pregnancies of the cohort mothers. The top value in each cell is the number of records with data recorded and the lower percentage shows the proportion of records with data recorded.

Total Records : 7637	Prev Pregs ==>									
VARIABLE	1	2	3	4	5	6	7	8	9	10+
**Gravidapreviouspreg**	7,637	3,965	1,824	760	327	152	71	28	9	11
	*100%*	*100%*	*100%*	*100%*	*100%*	*100%*	*100%*	*100%*	*100%*	*100%*
*{The gravida, or number of times the mother has been pregnant, at each recorded previous pregnancy}*										
**Noofbabiesprevpreg**	7,127	3,695	1,688	696	301	139	68	26	9	11
	*93%*	*93%*	*93%*	*92%*	*92%*	*91%*	*96%*	*93%*	*100%*	*100%*
*{The number of babies born at each recorded previous pregnancy}*										
**Currentstateofinfant**	4,236	2,404	1,058	413	180	87	32	16	6	8
	*55%*	*61%*	*58%*	*54%*	*55%*	*57%*	*45%*	*57%*	*67%*	*73%*
*{The well-being, if known, of each infant born at each recorded previous pregnancy}*										
**Prevpregdeliverymethod**	7,637	3,965	1,824	760	327	152	71	28	9	11
	*100%*	*100%*	*100%*	*100%*	*100%*	*100%*	*100%*	*100%*	*100%*	*100%*
*{The method of delivery for each infant of each recorded previous delivery}*										
**prevpregsexofbaby **	5,675	2,752	1,206	476	204	98	36	17	7	11
	*74%*	*69%*	*66%*	*63%*	*62%*	*64%*	*51%*	*61%*	*78%*	*100%*
*{Gender of each infant of each recorded previous delivery}*										
**Prevpregnancyoutcome**	5,884	2,892	1,297	521	221	98	36	16	6	8
	*77%*	*73%*	*71%*	*69%*	*68%*	*64%*	*51%*	*57%*	*67%*	*73%*
*{The outcome for each foetus of each recorded previous delivery}*										
**monthprevpregcompleted**	7,169	3,457	1,499	582	239	115	51	22	8	8
	*94%*	*87%*	*82%*	*77%*	*73%*	*76%*	*72%*	*79%*	*89%*	*73%*
*{The calendar month in which each recorded previous pregnancy came to an outcome}*										
**Yearprevpregcompleted**	7,637	3,965	1,824	760	327	152	71	28	9	11
	*100%*	*100%*	*100%*	*100%*	*100%*	*100%*	*100%*	*100%*	*100%*	*100%*
*{The calendar year in which each recorded previous pregnancy came to an outcome}*										
**Prevpregweightofbaby**	5,342	2,495	1,084	423	186	89	33	16	6	8
	*70%*	*63%*	*59%*	*56%*	*57%*	*59%*	*46%*	*57%*	*67%*	*73%*
*{Birth weight, grams, of each infant of each recorded previous delivery}*										
**Prevpregplaceofdelvry**	7,636	3,965	1,824	759	327	152	71	28	9	11
	*100%*	*100%*	*100%*	*100%*	*100%*	*100%*	*100%*	*100%*	*100%*	*100%*
*{Location, in free text, where each recorded previous pregnancy was delivered}*										
**Prevpregnancygestation**	7,321	3,769	1,712	706	304	145	64	28	9	11
	*96%*	*95%*	*94%*	*93%*	*93%*	*95%*	*90%*	*100%*	*100%*	*100%*
*{Gestation, in weeks, reported for each recorded previous pregnancy}*										
**prevpregcomplications**	607	380	185	73	38	19	6	2	1	0
	*8%*	*10%*	*10%*	*10%*	*12%*	*13%*	*8%*	*7%*	*11%*	*0%*
*{Free text description of the primary complications, if any, which were reported during each recorded previous pregnancy}*										
**otherprevpregcomps**	2,460	1,448	683	265	99	53	23	9	4	2
	*32%*	*37%*	*37%*	*35%*	*30%*	*35%*	*32%*	*32%*	*44%*	*18%*
*{Free text description of secondary or further complications, if any, which were reported during each recorded previous pregnancy}*										

The variables seen in Table 2, **gravidapreviouspreg, noofbabiesprevpreg, monthprevpregcompleted, yearprevpregcompleted, prevpregweightofbaby** and **prevpregnancygestation**, are all numeric values. The variables **prevpregplaceofdelvry, prevpregcomplications and otherprevpregcomps** are free text values and are currently not coded and potentially disclosive. Access to these free text variables is dependent on the availability of funding for processing.

The remaining variables only have a fixed set of categorical options available, as shown in Table 3.

**Table 3.** Categorical values of some variables available describing previously recorded pregnancies.

currentstateofinfant	prevpregdeliverymethod	prevpregsexofbaby	prevpregnancyoutcome
Deceased	Assisted Breech	Female	Ectopic Pregnancy
Neonatal death 1'st week	Breech Extraction	Male	Hydatidiform mole
Neonatal death 7–28 days	Elective C/S	Not Known	Livebirth
Not Known	Emergency C/S		Missed Abortion
Not Well	Low Forceps		Stillbirth
Well	Mid Cavity Forceps		Termination
	Not known		
	Other Unspecified		
	Rotational Forceps		
	Spont other cephalic		
	Spontaneous Vertex		
	Spontaneous miscarriage		
	Therapeutic Termination		
	Ventouse		

Delivery details –

Table 4 describes the level of completeness of data available from the 11,561 birth records of the G1 babies who were delivered at one of the two NHS sites for which data was available. This represents over 80% of the enrolled G1 cohort. Only those G1 babies with a birth, or neonatal treatment, recorded in Bristol at Southmead Hospital NHS Trust or St Michael’s Hospital NHS Trust and with either consent or section 251 permission to share health data are included. The level of completeness of the data only indicates how many records have some data recorded and does not indicate accuracy or quality of that data.

**Table 4.** Details of completeness and data type of the variables available describing the labour, delivery, birth and outcome of the cohort babies. Note *Vitamin K Administered* and *Vitamin K Given* are very similar but their values are not completely identical and so both variables are included here.

VARIABLE	# Completed	% Completed	Format
Rupt of Memb-Del Interval	10884	94.1%	hh:mm
Date of Rupt of Membranes	10884	94.1%	MMM-yyyy
Time of Rupt of Membranes	10884	94.1%	hh:mm
Presentation At Delivery	11561	100.0%	List
Method Of Delivery	11561	100.0%	List
Length of Labour 1st Stge	10315	89.2%	hh:mm
Length of Labour 2nd Stge	10315	89.2%	hh:mm
Reason for Abn Delivery 1	2509	21.7%	free text
Reason for Abn Delivery 2	604	5.2%	free text
Reason for Abn Delivery 3	77	0.7%	free text
Reason for Abn Delivery 4	10	0.1%	free text
Length of Labour 3rd Stge	11558	100.0%	hh:mm
Placental Weight	4934	42.7%	integer
Abnormal Placenta Details	1860	16.1%	free text
Time Of Birth Of Baby	11561	100.0%	hh:mm
Outcome	11561	100.0%	List
Baby's Date of Death	.	.%	MMM-yyyy
Baby's Sex	9806	84.8%	List
Baby's Birth Order	11561	100.0%	integer
Baby's Weight	11541	99.8%	integer
Birth Length	6646	57.5%	integer
Head Circumference	6683	57.8%	integer
Baby's First Breath	11508	99.5%	integer
Apgar Score At 1 Minute	11541	99.8%	integer
Apgar Score At 5 Minutes	11526	99.7%	integer
Resuscitation 1	11545	99.9%	List
Resuscitation 2	1034	8.9%	List
Resuscitation 3	234	2.0%	List
Resuscitation Drugs	249	2.2%	free text
Type Of Feeding	6594	57.0%	List
Vitamin K Administered	6594	57.0%	List
Hips	6594	57.0%	List
Baby Abnormality	4765	41.2%	free text
Postnatal Asses. of Gest.	5988	51.8%	integer
Bilirubin Monitoring	6613	57.2%	List
Level Of Bilirubin	1173	10.1%	integer
Special Care Factors 1	1634	14.1%	List
Special Care Factors 2	491	4.2%	List
Special Care Factors 3	231	2.0%	List
Special Care Factors 4	155	1.3%	List
Special Care Factors 5	110	1.0%	List
Special Care Factors 6	80	0.7%	List
Special Care Factors 7	56	0.5%	List
Special Care Factors 8	43	0.4%	List
Special Care Factors 9	26	0.2%	List
Special Care Factors 10	13	0.1%	List
Special Care Factors 11	5	0.0%	List
Special Care Factors 12	.	.%	List
Baby's Discharge Date	11279	97.6%	MMM-yyyy
Cord Complications	11561	100.0%	List
Placenta	11561	100.0%	List
Status of Person Cond Del	11557	100.0%	List
Sts of Sen Person Present	11557	100.0%	List
Paed. present at Delivery	11557	100.0%	List
Oth Reason for Abn Del 1	1215	10.5%	free text
Oth Reason for Abn Del 2	312	2.7%	free text
Oth Reason for Abn Del 3	72	0.6%	free text
Oth Reason for Abn Del 4	12	0.1%	free text
Date Of Birth Of Baby	11561	100.0%	MMM-yyyy
Time of Del of Placenta	11558	100.0%	hh:mm
Date of Del of Placenta	11558	100.0%	MMM-yyyy
Total Length Of Labour	10885	94.2%	hh:mm
Regular Respiration	11486	99.4%	integer
Vitamin K Given	11535	99.8%	List

Pregnancy details -

The table in the *Extended data*, Supplement I ^6^ as (the full table with details of completeness and data type of the variables available which describe the ante-natal, post-natal, maternal, pregnancy and treatment of the cohort mothers and babies is quite substantial, listing 406 variables, and so has been moved to the *Extended data* ^6^ as ‘Supplement I’) describes the level of completeness of data available from the 11,408 pregnancy records of the G0 mums in relation to the G1 babies who were subsequently delivered at one of the two NHS sites for which data was available. This represents 90% of the total G1 cohort. Only those G0 mums delivering at, and G1 babies with a birth recorded in or admitted as a neonate to, Bristol at Southmead Hospital NHS Trust or St Michael’s Hospital NHS Trust and where ALSPAC have permission to use health records are included. The level of completeness of the data only indicates how many records have some data recorded and does not indicate accuracy or quality of that data.

## Key STORK numbers

This summary of some of the main totals derived from the combined STORK dataset only includes those cases enrolled in ALSPAC and with either direct consent or section 251 permission. Note that some variables have some missing data and so not all totals or sub-totals are the same, for example the baby’s gender was only specified in 9,806 (85%) of the 11,561 records. Table 5 shows the number of births in the STORK data at the two NHS maternity trusts from which ALSPAC sourced this data. Table 6 details the recorded genders of the babies at the two NHS trusts, although as noted above the records are not fully complete. Table 7 shows the number of multiple pregnancies recorded at each of the NHS trusts. ALSPAC also has many other datasets which together with the STORK dataset may supplement each other, the section ‘Data availability’ below describes this process fully.

**Table 5.** Births of enrolled index participants of ALSPAC recorded within the dataset tabulated by NHS provider.

Dataset enrolled births by provider		
Southmead	6,771	
St Michael’s	4,790	11,561
Not in STORK data		2,501
Total		14,062

**Table 6.** Births of enrolled index participants of ALSPAC recorded within the dataset tabulated by NHS provider and gender.

Dataset enrolled live births by gender	Male	Female
Southmead	2,526	2,492
St Michael’s	2,430	2,358

**Table 7.** Births of enrolled index participants of ALSPAC recorded within the STORK dataset tabulated by NHS provider and whether singleton or multiple birth.

		Multiples (twins, triplets, etc.)
Multiple pregnancies listed in dataset	Singletons	
Southmead	6,603	84
St Michael’s	4,656	65

## Dataset validation

A detailed validation of the data was performed in January 1993, just after it was collected, on a sub-sample of 200 deliveries. This sub-sample was of 50 deliveries from each of the two trusts in each year 1991 and 1992, one of these deliveries was a set of twins. It is not detailed how these particular pregnancies were selected. The STORK data was carefully compared through direct examination to the abstracted obstetric and paediatric records provided by the trusts.

The STORK dataset was found to be less comprehensive and less complete than the abstracted records. Where data was present in both the STORK data and the abstracted ALSPAC obstetric and paediatric notes there was generally over 90% agreement between the two sources. The main areas of disagreement can be summarised as follows:

- Differences in what constituted a ‘booking visit’, such as GP, midwife or hospital antenatal clinic. Also, a mother may have had more than one of these and it is not clear which would have been indicated as ‘the’ booking visit. Differences in measurements, such as weight or haemoglobin level, taken at booking may also have similar issues with consistency because they may, in fact, refer to different visits.
- Differences in interpretation of the definitions of ‘pregnancy complications’, `perineum complications’ and ‘medical history’.
- Differences in what constitutes an ‘antenatal’ admission, and therefore differences in how many antenatal appointments.
- Variation of indicators of what signifies the beginning of labour influences how the length of labour, particularly stage 1, is measured.
- Discharge date may indicate simply discharge from the antenatal admission rather than discharge from the trust.
- Some data items were not recorded with any regularity, such as baby’s head circumference, administering of vitamin k, EDD by obstetrician, blood group.

In addition to those areas of disagreement noted above, the STORK dataset was less comprehensive in completed data regarding:

- Obstetricians’ estimated date of delivery (EDD) antenatally and the paediatrics’ postnatal assessment of gestation.
- Blood type and rhesus.
- Antenatal admissions and previous medical histories.
- Complications of the current pregnancy.
- Details of labour complications, induction and abnormal delivery.
- Fetal monitoring and also fetal distress during labour.
- Postnatal details of feeding, hip testing, length, head circumference and any abnormalities.

The report is reproduced in full in the *Extended data* ^6^ as ‘Supplement II’. The view of the report is that the paper abstracted ALSPAC obstetric and paediatric records were generally more reliable and more complete than the early STORK data. The abstracted records are only available as a full electronic dataset for specific detailed variables such as longitudinal measures of maternal weight and blood pressure, diagnosis of pre-eclampsia; Other details of pregnancy have been abstracted from the medical records for 8,369 pregnancies ^7^. The benefit of the STORK data is that it is readily available in an electronic format, although the accuracy of many of the variables is imperfect.

## Ethical approval

Ethical approval for the study was obtained from the ALSPAC Ethics and Law Committee and the Local Research Ethics Committees. A comprehensive list of research ethics committee approval references is available to download at: http://www.bristol.ac.uk/alspac/researchers/research-ethics/.

The initial approvals for the ALSPAC project were obtained as:

- Bristol and Weston Health Authority: E1808 Children of the Nineties: Avon Longitudinal Study of Pregnancy and Childhood (ALSPAC). (28th November 1989)
- Southmead Health Authority: 49/89 Children of the Nineties - "ALSPAC". (5th April 1990)
- Frenchay Health Authority: 90/8 Children of the Nineties. (28th June 1990)

The approval specifically for the ‘STORK’ dataset was sought and granted in May 1992 separately from the Bristol & District Health Authority and Southmead Health Services.

Permissions for the use of data collected via questionnaires and clinics and record linkage was based on the recommendations of the ALSPAC Ethics and Law Committee and NHS Research Ethics Committee’s at the time. Study participants have the right to withdraw their consent for elements of the study or from the study entirely at any time. Full details of the ALSPAC consent procedures are available on the study website ( http://www.bristol.ac.uk/alspac/researchers/research-ethics/).

## Data availability

### Underlying data

ALSPAC data access is through a system of managed open access. The steps below highlight how to apply for access to the data included in this data note and all other ALSPAC data:

1. Please read the ALSPAC access policy ( http://www.bristol.ac.uk/media-library/sites/alspac/documents/researchers/data-access/ALSPAC_Access_Policy.pdf) which describes the process of accessing the data and samples in detail, and outlines the costs associated with doing so.
2. You may also find it useful to browse our fully searchable research proposals database ( https://proposals.epi.bristol.ac.uk/?q=proposalSummaries), which lists all research projects that have been approved since April 2011.
3. Please submit your research proposal ( https://proposals.epi.bristol.ac.uk/) for consideration by the ALSPAC Executive Committee. You will receive a response within 10 working days to advise you whether your proposal has been approved.

The availability of our linked participant records is dependent on our ethical approvals and contractual arrangements with the NHS. If you are interested in using these data then please contact the ALSPAC Data Linkage Team ( alspac-linkage@bristol.ac.uk).

### Extended data

Open Science Framework: ALSPAC STORK. https://doi.org/10.17605/OSF.IO/MT4QF ^6^. 

This project contains the following extended data:

- Supplement_I: Table with details of completeness and data type of the variables available describing the ante-natal, post-natal, maternal, pregnancy and treatment of the cohort mothers and babies.
- Supplement_II: Notes to aid interpretation of the figures in the tables and the notes referring directly to the STORK data. These are not exhaustive but indicate of areas where differences in data collection for ALSPAC and STORK may account for the apparent differences in accuracy recorded.
- Supplement_III: Documentation describing the background of and data dictionary values for the South Western Regional Health Authority STORK Maternity System.

Data are available under the terms of the Creative Commons Attribution 4.0 International license (CC-BY 4.0).
