# Supplementary material for: The STORK dataset: Linked midwifery and delivery records of the mothers and index children in the Avon Longitudinal Study of Parents and Children (ALSPAC)
Source: Wellcome Open Res. 2020 Oct 5;5:229. [Version 1] doi: 10.12688/wellcomeopenres.16247.1 (PMC7726846; doi:10.12688/wellcomeopenres.16247.1)
Supplement: Peer review history

# Peer review: Reviewer response for version 1

**DOI:** 10.21956/wellcomeopenres.17845.r41580

**Published:** 2020-12-08

**Authors:** Ranadip Chowdhury

**Affiliations:** 1 Centre for Health Research and Development, Society for Applied Studies, New Delhi, India

1. The rationale for developing the STORK should be discussed in the introduction (i.e. how this dataset will add value to the existing ALSPAC). 
2. A tentative plan for utilization of the data should have been mentioned. 
3. A list of variables with their definitions and time points of assessments should have been included. 

Are sufficient details of methods and materials provided to allow replication by others?

Yes

Is the rationale for creating the dataset(s) clearly described?

Yes

Are the datasets clearly presented in a useable and accessible format?

Yes

Are the protocols appropriate and is the work technically sound?

Yes

Reviewer Expertise:

Maternal and Child nutrition

I confirm that I have read this submission and believe that I have an appropriate level of expertise to confirm that it is of an acceptable scientific standard.

# Peer review: Reviewer response for version 1

**DOI:** 10.21956/wellcomeopenres.17845.r40720

**Published:** 2020-10-28

**Authors:** Sally Bridges

**Affiliations:** 1 Born in Bradford, Bradford Institute for Health Research, Bradford Teaching Hospitals NHS Foundation Trust, Bradford, UK

This data note clearly describes the steps taken to link antenatal and delivery information from the STORK database with ALSPAC data. It describes the STORK database in a good level of detail and provides a clear rationale for linking these early electronic records. The validation work carried out is well described and the potential value of the linked dataset is discussed, including the limitations and unknown elements of it. Access to the dataset is clearly explained and the supplementary data is clear and accessible.

 There are a few minor comments below to follow up:

1. There is a discrepancy between the numbers listed in Figure 2 and those in the text (i.e. in Figure 2 total records =18454 and ineligible records =27, whereas in the text total records = 18,444 and ineligible records =17). 
2. Towards the end of 'The STORK database' section, the hospitals that used STORK are introduced. At the start of the following section, 'St Michael's records' is mentioned. It is not clear whether this is another term for one of the hospitals mentioned or something else. 
3. There is some inconsistency in the terminology used throughout (e.g. birth outcomes lists and delivery lists, child health database and child health system). I assume these are interchangeable but the authors may wish to change or clarify this. 
4. Section 251 is referred to in the note. The authors may wish to add a reference here explaining this, particularly for international readers.
5. Table 4 may benefit from some further labelling of some terms for clarity (e.g. Reason for Abn Delivery). 

Are sufficient details of methods and materials provided to allow replication by others?

Yes

Is the rationale for creating the dataset(s) clearly described?

Yes

Are the datasets clearly presented in a useable and accessible format?

Yes

Are the protocols appropriate and is the work technically sound?

Yes

Reviewer Expertise:

Birth cohort studies, data linkage

I confirm that I have read this submission and believe that I have an appropriate level of expertise to confirm that it is of an acceptable scientific standard.
